# Factors contributing to regional inequalities in acute respiratory infections symptoms among under-five children in Nigeria: a decomposition analysis

**DOI:** 10.1186/s12939-017-0626-7

**Published:** 2017-08-07

**Authors:** Oluwafunmilade A. Adesanya, Amadou Darboe, Bomar Mendez Rojas, Deji Emmanuel Abiodun, Idrissa Beogo

**Affiliations:** 1 International College of Medicine, Institute of Public Health, International Health Program, National Yang Ming University, Taipei, Taiwan, Republic of China; 2 University of Melbourne, Parkville, VIC Australia; 3 Centro de Investigaciones e Intervenciones en Salud, León, Nicaragua; 4 Business School, Department of Management, University of South Wales, Pontypridd, UK; 5 École Nationale de Santé Publique, Ouagadougou, Burkina Faso

**Keywords:** Inequalities, Nigeria, Children, ARI, Decomposition analysis

## Abstract

**Background:**

Acute respiratory infections (ARI) are major causes of morbidity and mortality in many low-income countries. Although factors associated with ARI symptoms in children under 5 years of age have been identified; however, variation in their prevalence resulting from regional-specific proximate determinants has received little attention. Therefore, we aim to investigate the specific regional determinants of overall and wealth-related inequality in children having ARI in Nigeria over a decade.

**Methods:**

We analyzed trends in development of ARI symptoms among children under 5 years of age in Nigeria using nationally representative cross sectional surveys carried out in 2003, 2008 and 2013. Overall- and household wealth index based- inequality in the distribution of prevalence of ARI symptoms were estimated by region using Gini index and Concentration Index, respectively. Multivariate logistic regressions for complex survey and decomposition analysis for both indexes were used to calculate percentual contribution.

**Results:**

We found a decreasing trend in development of ARI symptoms over the decade between regions. Children in South Western region had reduced likelihood of developing the symptoms. Concentration index (CI) for the prevalence of ARI symptoms over the years and across regions had negative values (all *p* < 0.05). Gini index (GI) varies from 0.21 in North East to 0.62 in South Western region. Furthermore, the mapping showed that the extent at which both inequalities contribute to ARI symptoms prevalence in each region is different. The four major sources of wealth-related inequalities were poor households, no maternal education, biomass cooking, and rural area.

The major contributors to overall inequalities were having a child aged 6 to 23 months, having no maternal education, having no vaccination card, and having a high birth order/short birth interval.

**Conclusions:**

Although ARI prevalence decreased over the decade, it has remained unequally distributed between regions and over the time. The sources of those inequalities are context sensitive. Thus, in future health promotion initiatives, it is imperative to account for regional variations in the distribution of ARI.

## Background

Acute respiratory infections (ARI) are the leading causes of morbidity and mortality in under five children [1, 2] as they account for over 2 million deaths and over 70% of common causes of clinical cases and hospitalization globally. [3, 4].. In addition, above half (i.e., 55%) of deaths due to ARI symptoms are from 15 low and middle income countries (LMIC)s [5] as nine of these countries are located in the sub-Saharan African (SSA) region. Nigeria accounts for the largest population in (SSA), of about 30,546,000 children under the age of 5 years [6]. ARI symptoms have been identified as the most significant determinants of morbidity and mortality in low-income countries [7], however, geographical location has seldom been considered as an explanatory factor for the large regional variations seen in childhood morbidity [8]. Preventive public health efforts (such as immunization programs) have been made to prevent and control ARI symptoms, but have progressed somewhat slowly, especially in Nigeria [9–11].

The International Vaccine Access Center (IVAC) developed an intervention score assessing the overall performance in adopting and implementing high impact strategies aiming at achieving the intervention target at 84% coverage by 2015. It is measured by the national rate of childhood vaccination, under-5 with suspected pneumonia receiving antibiotics, being taken to appropriate health care provider, and exclusive breastfeeding. Results show that Nigeria lags behind in achieving the goal, with its intervention score at 25 and 37% in 2013 and 2014, respectively, while Kenya had 57 and 65% in the same years [12, 13].

Nigeria, a home to the highest population in SSA region, constitutes of six major regional administrative boundaries with heterogeneity in terms of socio-cultural, geographic, political, economic, climate, religious, and ecological contexts [8, 14]. A population based study in Nigeria focusing on the spatial inequality used the mean logarithmic deviation to assess the magnitude of inequality; their findings revealed geographic location as a significant determinant of socio economic conditions [15]. In a spatial analysis of risk factors for childhood morbidity in Nigeria, Kandala et al. [8] observed spatial inequality in social and economic development between regions. For instance, the western region was used as a benchmark for free tuition and agricultural industrial development [8]. Consequently, individuals in this region have relatively better socioeconomic opportunities [16] and are likely to have better information about the risk factors for ARI symptoms than others, hence effectively reducing its prevalence. In addition, the Nigerian immunization coverage has been unequal across regions with the more developed regions in the south performing better compared with the less developed regions in the north [17, 18].

Differentials in child health outcomes existing between countries [19] may be attributed to within-country heterogenecity, requiring the need of tailored-context interventions to achieve effective and efficient prevention and control [8]. Meanwhile, the burden reveals longstanding sources of disadvantage and persistent inequalities. The Gini index for Nigeria in 2003 was 40.1, increased to 43.0 in 2010, and then to 48.8 in 2013, indicating higher inequality over time [20, 21]. The increase in socio-economic inequality may have impacted the distribution of household and regional-level determinants of child health outcomes [22]. These socio-economic inequalities in health persist amongst children from poorer regions and neighborhoods and having poorer households characteristics, being more likely to be exposed to conditions that exacerbate health outcomes, especially ARI [23, 24].

In order for the international community to achieve social justice in health for child health, the International Commission on Social Determinants of Health’s aim to understand and reduce health inequalities by reaching the mostly affected subpopulations [25]. Differential exposures of niche where a child is born and raised constitutes determinants of health outcomes [26]. Thus evidence of specific interventions effective in reducing ARI is necessary [27] for understanding the contributing factors to the inequity of ARI prevalence and examining why children from poor regions, with political problems and religious unrest in recent times leading to air pollutions, migration which may result into communities or households having higher risk of ARI [28].

Guided by the social determinants of health, “proximate determinants” are influenced by individual, household, and neighborhood patterns [29] with the argument of health outcome inequalities impact on child health outcome [23]. Higher odds of ARI symptoms are associated with children from households with mothers of low educational attainment and low wealth index [30, 31]. An International review on evidence of socio-economic inequalities carried out in LMICs reported that maternal education is strongly associated with childhood outcome within and between countries [23], indicating pathways in relation to health-related behaviors [32]. In regard to health related behaviors biomass cooking fuel, commonly use in households in LMICs has been investigated [33]. Gordon et al. carried out a systematic review on the association between household air pollution and respiratory infections and established a biological plausibility between children’s exposures to biomass cooking fuel and ARIs [34]. Other studies, including the Sonego et al.’s synthesized systematic review —on 39 LMICs— showed that chronically malnourished had 4 times higher risk of dying from ARI symptoms [35], and under-nutrition may be caused by socio-economic inequalities more than acute under-nutrition [36].

Using the World health organization (WHO) child growth standards, and measured socio-economic inequality using concentration index, Van de Poel ecological study including 47 countries contended that child malnutrition (stunting and wasting/thinning) disproportionately affected the poor [36]. Adesanya and Chiao in their findings revealed children living in the North western/eastern Nigeria had higher odds of ARI symptoms indicating inequalities in outcomes [28]. Still, no study has included these sources to explain the unequal distribution of ARI by region and over time especially in the Nigerian context.

Wealth index been an indicator of proximate effects linking household condition to influencing ARI symptoms, Van Malderen et al. using decomposition of Gini and Concentration index to investigate and compare the main determinants of overall inequality and wealth-related inequality in under-5 mortality in 13 African countries, revealed that the socioeconomic status, type and region of residence in Nigeria, independent of other factors, contributed substantially to reflecting inequality in under five mortality [37]. This trend has not been considered in examining ARI symptoms, as it is imperative to understand how time period changes have resulted in varying effects of ARI symptoms over the last decade in order to improve health intervention outcomes. Therefore, we extend the research line by employing the use of regression based decomposition analysis [38], which allows an estimation of the relative contribution of each predictor on both wealth-related and overall inequalities. A similar analytical strategy has been used for prior studies that identified the sources of wealth-based inequalities on self-rated health [39], poor mental health [40], children’s malnutrition [36] and infant mortality [41].

We hypothesize that using this analytical strategy will allow us to clearly identify the effects of specific predictors of children developing ARI on ARI wealth based inequalities. We further hypothesize that the sources of wealth-related and overall inequalities in the distribution of children’s symptoms of ARI will differ by region and over time.

Identifying and focusing on varying contributing factors on child health outcomes [19] specific to each region may in turn help towards the effective reduction of ARI [8]. Since Nigeria is identified as a major contributor to the global child mortality [42] primarily due to ARI, this needs to be reduced in order to work towards achieving the sustainable development goals set by the international community to reduce under 5 mortality to as low as 25 out of 1000 live births by 2030 [31].

## Methods

### Study design and sampling technique

We used the three latest nationwide representative cross-sectional surveys carried out in Nigeria in the years 2003, 2008 and 2013. The surveys employed a national probability sample of households using a two-stage cluster sampling technique. The country characterized by six-geopolitical zones was stratified into 36 states and the Capital Abuja, summing to 37 districts. The primary sample unit (PSU), which was regarded as a cluster for NDHS, in the first stage was derived from the prior Nigerian population census enumeration areas (EAs). Further details can be found at [9–11, 43]. Our analytical sample is composed of children aged below 5 years: 5159 in 2003 [9], 25,199 in 2008, [10] and 28, 596 in 2013 [11].

### Ethical approval

This study employed the use of secondary analysis of an already collated dataset, as the survey personnel obtained ethical approval from the National Ethic committee of the Federal Ministry of Health, Abuja, Nigeria and ICF international, Rockville, MD, USA, with the following dataset online for accessibility. This proposed research has also been approved by the Institutional Review Board of National Yang-Ming University.

### Operationalization of the major measures

#### Outcome variable

The dependent variable used in this study was symptoms of ARI [9–11]. All mothers of children below 5 years were asked whether their children had been ill with a cough in the 2 weeks preceding the survey. For children who had a cough, the mother was additionally asked if the child’s cough was accompanied by short, rapid breathing and fever during the last 2 weeks prior to the survey. The outcome is a binary variable where 1 is children who met all mentioned criteria and 0 otherwise.

#### Individual and household covariates

We included a set of variables known to predict ARI symptoms: child’s gender (male or female), child’s age (in months), cooking methods (kerosene, biomass and others), maternal education (No education, primary, secondary and higher education) household wealth index (poorest, poorer, middle richer, richest), type of residence (rural or urban), and child health card (evidence to the interviewer on coverage of age specific vaccination interventions, vitamin A, growth monitoring) [44]. We also included whether the child received BCG vaccine at birth i.e., yes, no, do not know [44, 45]. The variable height/age reflects chronic malnutrition and has three categories: growth deficit (less than -2SD), normal (between −2 SD and 2 SD) and above normal (greater than 2 SD) [46]. For the variable “birth order” and “interval”, the category “short” signifies preceding birth intervals less than 24 months and “long” denotes preceding birth intervals greater than 24 months [37]; further short birth intervals and birth order 2 to 4 are considered *risky birth intervals* [37]. Those cut-off points were selected based on prior research that have identified a high occurrence of adverse events for intervals shorter than 24 months [47]. Region is a six-level variable (North west, North east, North central, South east, South south and South west) that was included to take into account between region heterogeneity [28].

### Data analysis

We used both descriptive and logistic regression for complex survey were carried out to assess determinants of ARI symptoms. We ensured national representativeness; weighting factors were employed in this study to adjust for over sampling of some areas and under-sampling of others in the Demographic and Health Survey by using sampling weights using Taylor series linearization method [48, 49]. Structurally, two indicators of inequality in the distribution of ARI symptoms namely Gini index [50] and Concentration index [51] were estimated. Gini index measures how much the population’s distribution of ARI symptoms deviates from perfect distribution (ie., perfect equality), with higher value of Gini (i.e., a value closer to 1) representing higher deviation and inequality [50]. Concentration index is calculated using household wealth index to quantify the degree ARI is unequally distributed [50, 51]. Concentration index ranges from −1 to +1 and more negative values represent higher health outcome (ARI symptoms) concentration among the lowest socioeconomic strata. Tertiles of Gini- and Concentration- index were estimated by region and year and displayed in maps.

Descriptive statistics were estimated to show between-region differences. In the multivariate logistic regression no selection of predictors was needed because our goal was to estimate predicted logs for all predictors. No evidence of multi-collinearity was found, with all variance inflation factors less than 10 [52]. In order to quantify how much of variation in both indexes can be attributed to each predictor, we performed a regression-based decomposition [53] using Wagstaff’s method for Concentration index [51] and Fields methods [54] for Gini index; such variation is interpreted as the percentual contribution of each predictor to wealth related or overall inequalities: the higher the value, the higher the contribution. The residual denote the levels of unexplained variation in Concentration Index: negative values indicate that predictors entered in decomposition analysis explain all wealth based inequality; positive values indicates the degree of Concentration index that remained unexplained given the predictors in the model. To meet the linearity assumption of this analytical approach, we decomposed the predicted logs of having ARI symptoms (obtained from logistic regression models) [37]. Our analysis for this current study was restricted to “alive children” born within 5 years preceding the survey in the 2003, 2008 and 2013 data set with the purpose to obtain a vivid picture of the current situations occurring in the different geo-political regions of the country. Also for the quality of the data collection by the NDHS, in their report they indicated that the percentage of missing information regarding birth, deaths, birth dates and age at death across various maternal characteristics and region of residence only varied between less than 1 to 3% [9–11]. Descriptive analyses, logistic regression and Gini Index were estimated in Stata version 12.0. Estimation of concentration index and decomposition analyses were estimated in R version 3.2.0 using the *decomp* package. Statistical significant level was set up at 0.05.

## Results

Table 1 portrays descriptive statistics by year and region. The prevalence of ARI symptoms has dropped across the years and ranges from 0.8 to 16.2. North east has the highest figures in the 3 years: 16.2%, in 2003, 7.54% in 2008 and 5.1% in 2013. South west has the lowest prevalence in the last two surveys. The proportion of households in the lowest quintile (i.e., poorest) declined over time in south-south and south-western region (percentages) and increased in north west and north eastern regions: north west (20.6% in 2003 and 37.1% in 2013), north east (34.9% in 2003 and 39.4% in 2013) and north central (19.9% in 2003 and 12.3% in 2013). The prevalence of ARI by each covariate is presented in the Appendix.

**Table 1:** Descriptive statistics of ARI and determinants by region, DHS 2003, 2008 and 2013 Nigeria

	North West			North East			North Central			South East			South South			South West		
Variables	2003 *N* = 1517%wt^a^	2008 *N* = 6772%wt^a^	2013 *N* = 8760%wt^a^	2003 *N* = 1234%wt^a^	2008 *N* = 5699%wt^a^	2013 *N* = 5856%wt^a^	2003 *N* = 893%wt^a^	2008 *N* = 4505%wt^a^	2013 *N* = 4286%wt^a^	2003 *N* = 460%wt^a^	2008 *N* = 2160%wt^a^	2013 *N* = 2553%wt^a^	2003 *N* = 482%wt^a^	2008 *N* = 2979%wt^a^	2013 *N* = 3498%wt^a^	2003 *N* = 573%wt^a^	2008 *N* = 3084%wt^a^	2013 *N* = 3643%wt^a^
ARI prevalence (%)	8.9	1.9	0.9	16.2	7.5	5.1	6.8	1.4	2.06	6.4	1.8	2.1	12.2	3.5	1.7	6.8	1.0	0.8
Child’s gender (female)																		
Female	48.9	49.7	50.7	47.8	50	48.5	49.6	49.2	49.7	56.2	48.8	49.4	50.2	48.9	49.9	45.1	49.1	49.6
Male	51.0	50.2	49.2	52.1	49.9	51.4	50.3	50.7	50.2	43.7	51.1	50.5	49.7	51.01	50.1	54.8	50.8	50.3
Child’s age (months)																		
0–5	12.8	10.9	9.8	12.2	12.5	10.7	11.4	11.3	10.7	10.2	11.4	10.5	12.7	12.1	10.3	13.9	11.4	10.2
6–11	12.2	11.9	11.8	12.5	10.4	10.5	14.2	11.5	10.1	7.51	10.9	12.2	12.6	11.6	11.1	14.2	11.9	11.4
12–23	19.6	20.3	20.0	17.9	19.6	20.3	19.1	18.6	20.2	21.2	20.7	21.2	17.6	20.1	21.5	16.6	19.2	20.1
24–35	18.6	18.5	18.3	18.8	18.4	18.7	18.9	18.4	19.8	28.3	19.4	19.1	21.7	19.1	20.1	17	18.4	18.8
36–59	36.5	38.2	39.8	38.3	38.8	39.5	36.2	40.0	39.1	32.6	37.4	36.7	35.2	37.1	36.8	38.1	38.8	39.2
Birth order and interval																		
1st birth	18.1	16.3	16.01	16.8	15.3	16.8	20.9	18.6	20.8	24.1	22.2	22.6	24.0	22.3	25.5	26.9	24.9	23.6
2 to 4 and short	10.0	9.9	8.8	11.8	9.7	11.2	10.1	8.9	9.4	19.8	17.8	16.4	8.2	12.1	11.5	9.85	8.9	10.2
2 to 4 and long	32.3	32.9	31.9	29.9	32.3	32.5	36.9	39.5	39.7	29.1	30.2	31.9	32.02	36.2	35.9	43.8	47.2	43.4
5 to + and short	8.6	8.95	9.4	9.9	9.9	9.3	4.62	5.8	5.3	6.5	7.6	6.3	6.0	6.3	5.3	1.3	2.7	3.2
5 to + and long	30.9	31.7	33.8	31.4	32.7	30.1	27.3	27.1	24.8	20.5	22.0	22.7	29.6	23	21.7	17.9	16.1	16.5
Height/Age (chronic)																		
Deficit	55.7	49.3	51.2	43.4	45	37.5	32.1	39.1	25.7	19.5	17.5	11.9	17.3	26.2	14.9	24.6	26.6	17.2
Normal	42.2	45.2	46.2	53.6	49.7	57.4	64.7	56.4	69.4	72.2	74.6	82.4	76.6	69.2	76.3	72.8	68.2	78.5
Above normal	1.96	5.39	2.5	2.9	5.19	5.07	3.2	4.4	4.8	8.2	7.8	5.6	6.0	4.5	8.7	2.5	5.1	4.2
Received BCG																		
No	74.5	80.2	77.8	63.9	73.6	65.1	40.8	40.8	41.1	13.9	21.1	13.3	28.5	25.2	18.7	13.5	18.4	17.6
DK	0	0.5	0.1	2.2	0.2	0.2	0.7	1.1	0.4	2.3	0.5	0.5	1.9	0.7	0.2	0.3	0.6	0.1
Child has health Card																		
Yes (seen/no seen)	17.7	16.9	20.7	29.4	25.5	34.6	50.3	49.0	50.5	84.8	79.3	83.3	56.3	69.8	75.9	78.6	61.8	80.8
No longer has card	2.8	1.8	1.5	0.8	4.0	1.57	5.5	3.6	4.7	2.3	1.2	1.3	4.9	4.2	4.3	5.7	18.8	0.4
Household Characteristics																		
Cooking methods																		
Kerosene	10.5	5.4	6.9	2.8	2.5	7.4	11.9	11.9	14.1	58.9	28.1	30.1	28.1	31.1	35.5	67.3	58.8	66.1
Biomass	88.5	93.9	92.7	95.9	96.9	91.6	85.7	85.9	83.1	38.8	69.5	67.5	64.8	64.3	57.3	27.4	38.2	28.6
Others	0.9	0.6	0.4	1.3	0.6	0.9	2.2	2.1	2.7	2.2	2.3	2.3	6.9	4.5	7.2	5.2	2.8	5.1
Maternal education																		
No education	73.1	78.0	76.2	71.1	73.2	70.2	41.2	43.6	41.4	7.2	6.9	4.5	9.9	6.5	7.19	12.1	14.5	10.6
Primary	13.1	13.1	12.3	16.8	16.5	14.2	36.0	29.5	25.8	32.1	31.1	27.1	38	35.3	28.1	31.8	28.4	25.4
Secondary	12.1	7.3	10.1	10.4	9.06	12.8	19.9	21.5	26.0	48.3	50.2	57.6	45.5	49.8	53.5	47.6	44.2	48.7
Higher	1.5	1.46	1.3	1.5	1.21	2.63	2.98	5.37	6.77	12.2	11.6	10.7	6.5	8.3	11.1	8.37	12.8	15.1
Household wealth index																		
Poorest	20.6	31.9	37.1	34.9	46.4	39.4	19.9	23.3	12.3	7.8	5.3	6.0	18.0	7.1	0.4	9.9	4.8	2.2
Poorer	27.2	32.7	31.1	24.3	24.2	27.4	18.5	24.5	22.6	11.7	10.7	12.7	15.4	16.3	10.7	6.5	11.1	7.6
Middle	22.8	17.8	15.1	20.2	16.3	15.5	26.9	24.9	32.3	14.3	25.0	23.5	16.8	22.5	27.0	6.9	13.3	12.5
Richer	19.5	11.2	10.6	15.4	10.0	11.3	22.4	15.3	19.8	12.9	31.0	28.0	24.8	28.1	31.7	18.4	25.2	28
Richest	9.72	6.2	5.9	5.1	2.9	6.2	12.1	11.8	12.9	53.1	27.8	29.0	24.8	25.9	30.1	58.1	45.5	49.7
Type of residence																		
Urban	25.3	18.2	23.6	25.2	26.2	25.4	21.4	25.2	22.7	49.3	46.1	69.9	25.3	27.6	35.2	69.1	55.8	72.1
Rural	74.6	81.7	76.3	74.7	73.8	74.5	78.5	74.7	77.2	50.6	53.8	30.0	74.6	72.3	64.7	30.8	44.1	27.8

^a^Unweighted N’s and weighted percentages and means are reported. Percentages may not add up to 100 due to rounding

### What are the determinants of ARI symptoms by region between 2003 and 2013?

Table 2 shows the logistic regression for the three consecutive years controlling for all potential predictors of ARI symptoms in children. In 2003, net of all variables for children living in the North Eastern region (OR = 2.75; *p* < 0.05) and Southern South region (OR = 1.79; *P* < 0.05) were associated with higher risk of developing ARI symptoms. Also, children from richest household quintile were found to be 51% less likely to have ARI. In 2008, children living in North Eastern region (OR = 5.91; *P* < 0.05), Southern region (OR = 3.10; *P* < 0.05) were found to have higher risk of ARI symptoms. Also, children aged 6–11 months (OR = 1.57; *P* < 0.05) and 12–23 months (OR = 1.49; *P* < 0.05) were found to have a higher risk of ARI. Furthermore, children with normal growth (OR = 0.81; *P* < 0.05) were 19% less likely to have ARI symptoms. Children immunized with BCG were 33% less likely to have ARI symptoms and children who no longer have health card were 32% less likely to have ARI symptoms. Children from poorer households (OR = 1.30; *P* < 0.05) were 30% more likely to have ARI symptoms.

**Table 2:** Multivariate logistic regressions of ARI between regions; regression coefficients and significance Demographic and Health Survey 2003, 2008 and 2013

Variables	2003 aOR (95% C.I)	2008 aOR (95% C.I)	2013 aOR (95% C.I)
Child’s residential characteristics			
Region			
South West	1	1	1
North central	0.91 (0.51, 1.63)	1.22 (0.70, 2.14)	2.31 (1.25, 4.26)**
North east	2.75 (1.58, 4.79)***	5.91 (3.51, 9.96)***	8.51(4.72, 15.3)***
North west	1.27 (0.72, 2.23)	1.51 (0.87, 2.62)	1.31 (0.69, 2.48)
South East	1.27 (0.68, 2.35)	1.63 (0.88, 3.02)	2.87 (1.52, 5.40)**
South South	1.79 (0.99, 3.24)**	3.10 (1.83, 5.25)***	2.20(1.17, 4.13)**
Urban rural			
Urban	1	1	1
Rural	1.25 (0.89-, 1.74)	0.99 (0.72, 1.36)	0.96 (0.70, 1.33)
Cooking methods			
Kerosene/Charcoal	1	1	1
Biomass	0.75 (0.46, 1.21)	0.83 (0.56, 1.24)	1.69(1.05, 2.71)**
Others	0.66 (0.27, 1.58)	0.26 (0.06, 1.13)	0.92 (0 .34, 2.45)
Child Characteristics			
Child’s gender (female)			
Male	1	1	1
Female	0.94 (0.76, 1.16)	1.06 (0.89, 1.27)	0.96 (0.80, 1.15)
Child’s age (months)			
0–5	1	1	1
6–11	1.37 (0.92, 2.04)	1.57 (1.08, 2.29)**	1.85 (1.25, 2.73)**
12–23	1.15 (0.77, 1.70)	1.47 (1.03, 2.10)**	1.95 (1.36, 2.80)***
24–35	0.93 (0.62, 1.39)	1.27 (0.88, 1.82)	1.40 (0.96, 2.04)
36–59	0.65 (0.45, 0.95)	0.93 (0.67, 1.31)	0.70 (0.48, 1.01)
Birth order and interval			
1st birth	1	1	1
2 to 4 and short	1.10 (0.75, 1.64)	0.70 (0.47, 1.03)	0 .81 (0.54, 1.19)
2 to 4 and long	0.84 (0.62, 1.14)	0.92 (0 .71, 1.20)	1.07 (0.81, 1.40)
5 to + and short	0.70 (0.43, 1.15)	1.08 (0.76, 1.55)	1.26 (0.85, 1.85)
5 to + and long	0.85 (0.62, 1.16)	1.03 (0.78, 1.35)	1.19 (0.90, 1.58)
Height/Age (chronic)			
Deficit	1	1	1
Normal	1.17 (0.92, 1.49)	0.81 (0.66, 0 .98)**	0.97 (0.79, 1.20)
Above normal	1.15 (0.63, 2.11)	1.13 (0.76, 1.68)	0.48 (0.25, 0.91)**
BCG			
Received	1	1	1
No	0.95 (0.68, 1.32)	0.67 (0.51, 0 .86) **	1.0 (0.75, 1.35)
Don’t know	0.73 (0.21, 2.63)	0.57 (0.13, 2.51)	2.04 (0.45, 9.17)
Child has health Card			
No card	1	1	1
Yes (seen/no seen)	1.11 (0.79, 1.57)	0.72 (0.43–1.18)	1.26 (0.93, 1.71)
No longer has card	1.38 (0.73, 2.59)	0.68 (0.51, 0.90)**	1.12 (0.57, 2.19)
Maternal education			
No education	1	1	1
Primary	1.06 (0.78, 1.43)	1.08 (0.84, 1.39)	1.24 (0.95, 1.63)
Secondary	0.77 (0.53, 1.12)	0.95 (0.68, 1.31)	1.69 (1.25, 2.30)**
Higher	1.07 (0.55, 2.09)	0.83 (0.42, 1.65)	1.35 (0.73, 2.49)
Household wealth index			
Poorest	1	1	1
Poorer	0.92 (0.66, 1.28)	1.30 (1.01, 1.68)**	1.18 (0.91, 1.55)
Middle	1.00 (0.70, 1.43)	0.96 (0.70, 1.32)	0.91 (0.65, 1.27)
Richer	0.84 (0.55, 1.28)	0.95 (0.64, 1.39)	0.58 (0.38, 0.88)**
Richest	0.49 (0.27, 0.93)**	0.63 (0.36, 1.13)	0 .48 (0.27, 0.87)**
Outcome measure			
Symptoms of ARI			

All odds ratios were estimated taking into account sampling weights

*CI* confidence interval, *aOR* adjusted odds ratios

*p* < 0.05^**^, 0.001^***^

In 2013, children residing in North central region (OR = 2.31; *P* < 0.05), North eastern region (OR = 8.51; *P* < 0.05), South eastern region, (OR = 2.87; *P* < 0.05) and Southern region (OR = 2.20; *P* < 0.05) were associated with having higher risk of ARI symptoms than children from the South western region. Children from households using biomass fuel (OR = 1.79; *P* < 0.05) were 69% more likely to develop ARI symptoms than children from households using kerosene/charcoal. Children aged 6–11 months (OR = 1.85; *P* < 0.05) and 12–23 months (OR = 1.95; *P* < 0.05) were found to have a higher risk of developing ARI symptoms than children between ages 0–5 months. Children with normal growth (OR = 0.48; *P* < 0.05) are 52% less likely to have symptoms of ARI. Children from mothers who have a secondary diploma (OR = 1.69; *P* < 0.05) were 69% more likely to have symptoms of ARI. Children from rich quintile households (OR = 0.58; *P* < 0.05) and richest quintile household (OR = 0.48; *P* < 0.05) were found to have a lower risk of developing ARI symptoms.

Table 3 shows the share represented for each variable to wealth-related inequalities measured by concentration index by region and year. The concentration index ranges from −0.24 (in south eastern region) to 0.13 (in south western region) and was significantly different from zero across all regions and years (except in 2008 for north eastern region) with concentration index of (−0.0006). The lower values for concentration index (i.e., a wider gap in the distribution of ARI symptoms between the poor and the rich) were found in south eastern regions.

**Table 3:** Relative contribution (%) of determinants to wealth related inequality (C) in likelihood of having ARI in Nigeria

Region	Concentration index (C)	C 95% confidence interval	Child’s gender (female)	Child’s age (6 to 23 months)	Birth order and interval (2 to 4 short)	Height/Age (chronic) (deficit)	No received BCG	Child has no health card	Cooking methods (biomass/others)	No maternal education	Household wealth index (poorest)	Type of residence (rural)	Residual
North West													
2003	−0.04**	−0.05, 0.03	0.04	−25.95	−0.23	−0.26	−0.82	0.63	4.56	8.49	112.25	1.79	−0.40
2008	−0.06**	−0.07, −0.05	−0.46	−7.75	6.23	1.50	−1.53	0.70	−12.10	40.40	79.42	−7.05	0.48
2013	−0.09**	−0.10, −0.09	0.005	0.388	0.077	−0.115	0.012	0.05	−0.04	−18.64	118.29	−0.25	−0.44
North East													
2003	0.02**	0.01, 0.03	2.36	19.72	−0.32	0.94	8.55	−1.46	−9.96	248.49	−38.40	−130.0	0.38
2008	−0.0006	−0.006, 0.005	0.92	8.90	105.32	−0.53	−1.38	5.57	−332.10	117.10	188.40	2.86	5.22
2013	−0.014**	−0.02, −0.006	1.86	−53.56	55.37	2.50	1.41	−3.29	−0.34	−269.4	284.10	80.9	0.61
North Central													
2003	−0.055**	−0.06, −0.05	−1.74	−12.55	0.80	−8.39	−4.67	2.52	30.54	13.27	20.05	61.00	−1.02
2008	−0.05**	−0.07, −0.03	0.36	0.64	26.45	9.32	−157.40	242.71	236.20	−107.70	−276.00	122.5	2.83
2013	−0.14**	−0.15, −0.12	−0.32	−3.77	−0.85	−7.02	0.67	−1.62	81.61	−69.09	117.09	−16	−0.83
South East													
2003	−0.2**	−0.25, −0.16	−1.09	−4.21	2.21	−2.99	2.76	−0.4	−9.65	69.30	45.17	−0.78	−0.33
2008	−0.24**	−0.27, −0.22	−1.19	0.21	−9.41	−2.70	−0.31	3.71	33.40	49.60	46.81	−20.60	0.34
2013	−0.14**	−0.16, −0.12	−0.18	−0.91	17.91	0.03	−0.24	−0.53	50.70	−97.97	127.90	−0.41	3.62
South South													
2003	−0.13**	−0.17, −0.09	−1.42	−4.26	−15.02	17.55	−2.66	1.87	−141.8	68.21	142.48	34.70	0.15
2008	−0.14**	−0.16, −0.13	0.71	−0.75	8.70	6.34	0.566	−0.80	9.65	−6.60	91.80	−9.76	0.03
2013	−0.14**	−0.17, −0.12	0.17	−4.24	−5.70	24.00	−0.63	−0.88	27.90	−65.50	130.50	−3.42	−2.00
South West													
2003	−0.19**	−0.24, −0.14	0.33	−2.07	−2.19	−16.20	−3.71	−2.83	9.20	28.8	−11.9	100.5	−0.10
2008	−0.05**	−0.06, −0.03	3.92	−3.96	3.64	−2.49	−3.79	0.75	−415.80	195.90	319.5	−0.03	2.10
2013	0.13**	0.10, 0.17	−0.01	2.28	13.08	0.67	−2.67	6.09	−255.70	−10.70	356.35	−4.52	−5.02

The relative contributions were estimated using decomp package in R [67]

*P* < 0.05**

During the three DHS survey waves, most socio-economic-related inequality observed across the regions were due to differences in household wealth index and maternal education. The only exception was in the north central, where cooking methods are an important contributor along with household wealth index. For example, in south eastern region in 2003, maternal education and household wealth index contributed to 69.3 and 45.17% of differences in wealth-based inequalities respectively. By 2013, cooking methods explained 81.61% of differences in wealth-based inequalities in north central. The unexplained contribution to wealth-related inequalities (residuals) ranges from −5.02 in South Western region to 5.22 in North Eastern region, the value in the latter region indicates that predictors explain 94.78% of total wealth based inequality.

### What are the sources of overall inequality in the distribution of ARI symptoms?

Table 4 displays overall inequality (Gini index) by region and year. Gini index ranges from 0.21 to 0.62, displaying an upward trend in all regions. For example, south eastern region increased from 0.38 in 2003 to 0.56 in 2013. North West and North Eastern regions had the lowest Gini index, which implies that children’s likelihood of having ARI symptoms are evenly distributed. On the other hand, South West and South Eastern regions had the highest Gini index, which indicates likelihoods of ARI symptoms are unequally distributed among subgroups of children. By the year 2003, differences in children’s age, maternal education, and type of residence were the main contributors to overall inequality. However, in 2008 and 2013, overall inequality is mainly explained by child’s age, birth order and interval, child’s nutritional status, cooking fuel, and maternal education. For example, by 2003 in South South region, differences in child’s age, maternal education and type of residence contributed to 14.1, 18.06, and 26.6% to overall inequalities, respectively.

**Table 4:** Relative contribution (%) of determinants to overall inequality in likelihood of having ARI in Nigeria

Region	Gini index	Child’s gender (female)	Child’s age (6 to 23 months)	Birth order and interval (2 to 4 short)	Height/Age (chronic) (deficit)	No received BCG	Child has no health card	Cooking methods (biomass)	Maternal education (no education)	Household wealth index	Type of residence
										(poorest)	(rural)
North West											
2003	0.32	0.12	43.60	11.02	3.55	21.5	7.23	8.49	2.0	−0.008	1.50
2008	0.31	7.17	13.10	5.40	1.18	47.1	−1.62	0.09	22.07	1.37	3.97
2013	0.34	27.2	36.3	3.51	−0.03	0.12	−0.10	−0.29	4.67	7.32	21.1
North East											
2003	0.22	0.70	24.8	10.5	33.9	4.33	−2.16	10.05	10.48	7.37	−0.06
2008	0.21	1.17	27.7	0.11	11.8	3.48	23.3	27.20	0.27	4.73	−0.04
2013	0.28	0.94	78.9	0.57	0.05	0.09	0.04	0.01	8.97	4.82	5.57
North Central											
2003	0.35	4.45	26.1	1.18	2.39	17.5	−0.02	10.2	5.67	10.79	21.5
2008	0.37	0.05	0.90	5.71	0.04	8.92	38.5	26.7	4.45	5.08	9.51
2013	0.51	0.77	32.3	2.89	1.4	0.24	0.18	30.5	23.6	3.75	4.19
South East											
2003	0.38	0.15	0.03	3.11	−0.42	0.39	4.13	1.2	76.09	16.4	−1.13
2008	0.51	11.00	28.9	3.31	0.97	7.76	1.05	16.9	29.8	−0.47	0.63
2013	0.56	0.07	50.10	8.00	7.42	0.68	−0.06	21.03	2.68	7.51	2.44
South South											
2003	0.43	−0.1	14.1	0.02	20.7	8.4	17.02	−3.32	18.06	−1.60	26.6
2008	0.33	2.15	6.81	16.30	14.6	1.35	20.30	12.20	16.10	11.6	−1.72
2013	0.55	2.74	56.1	33.30	−0.005	−0.01	0.02	0.004	7.26	0.01	0.46
South West											
2003	0.47	0.36	13.9	−0.05	2.24	36.6	0.47	−0.19	−2.46	4.44	44.6
2008	0.58	7.27	6.44	6.77	6.23	−0.54	8.80	2.88	56.6	2.88	2.55
2013	0.62	0.21	11.2	44.2	1.92	−1.18	26.1	9.57	5.34	1.49	1.01

The relative contributions were estimated using Fields methods [54]

Further, in the year 2013, differences in child’s age, cooking methods, birth interval and order, household wealth index and child’s nutritional status contributed to 50.1, 21.0, 8.0, 7.5 and 7.42%, respectively to overall inequality in South Eastern region.

### Map showing tertile variation in Gini index, concentration index, and ARI symptoms between; 2003–2013

According to the current political administrative policy, Nigeria is divided into 6 major geo-political regions namely; South West (SW), South South (SS), South East (SE), North Central (NC), North East (NE) and North West (NW). With ranges grouped into tertiles, the above figure shows the contribution of determinants to the overall inequality in the distribution of ARI symptoms measured by Gini index (GI) and wealth related inequality in ARI symptoms measured by the concentration index (CI) over the decade. Concentration index was negative over the years and within all regions, which indicate an excessive concentration of ARI symptoms among low SES strata. However, the level of this concentration differs across regions, with regions in the first tertile showing a wider gap than those in the second and third tertile.

In 2003, the ***first map*** (blue) represents the GI for the region. The (NW) and (NE) regions are in the first tertile, indicating low inequality in the distribution of ARI symptoms within the regions. The regions (NC) and (SE) are in the second tertile, indicating some inequality in the distribution of ARI symptoms. The (SW) and (SS) regions were observed to be within the third tertile, indicating high inequality in the distribution of ARI symptoms within those regions. Moving forward, the ***second map*** (green) describes the CI for each region. The (SW) and (SE) regions were within the first tertile of CI, indicating ARI symptoms are highly concentrated within low socioeconomic strata.

Regions (NC) and (SS) were within the second tertile, indicating a Medium marked concentration of ARI symptoms within the low socioeconomic strata. In comparison, (NC) and (NW) regions were in the third tertile, indicating lower levels of wealth-based inequalities in the distribution of ARI symptoms. The ***third map*** (red) represents the prevalence of ARI symptoms, showing (NC) within the first tertile, indicating low prevalence. (NW) and (SW) region were within the second tertile, indicating a medium level prevalence of ARI symptoms within the two regions, while (NE), (SS), and (SE) regions were within the third tertile, indicating the highest prevalence of ARI symptoms in the three regions for 2003.

In 2008, the ***first map*** (blue) represents the Gini for the region. The (NW) and (NE) regions were within the lowest tertile, indicating low inequality in the distribution of ARI symptoms. The (NC) and (SS) regions were within the second tertile, showing some inequality in distribution. The (SW) and (SE) regions were within the highest tertile, corresponding to higher unequal distribution of ARI symptoms within those regions. The ***second map*** (green) describing the CI for each region showed the (SS) and (SE) regions within the lowest tertile, indicating ARI symptoms are highly concentrated within the low SES groups. Regions (NC) and (NW) were within the second tertile, indicating a medium range of ARI symptoms concentrating within the low SES groups while (SW) and (NE) regions were in the highest tertile, indicating lower levels of wealth-based inequalities in the distribution of ARI symptoms. Furthermore, the ***third map*** (red) describes the prevalence of ARI across regions in 2008. Regions (NC) and (SE) were within the first tertile, indicating a low prevalence of ARI symptoms, while (NW) and (SE) regions were within the second tertile with a slightly elevated prevalence of ARI symptoms. Regions (NE) and (SS) were within the third tertile, suggesting that the two regions had the highest prevalence of ARI symptoms in 2008.

In 2013, the ***first map*** (blue) represents the GI for the year. The (NE) and (NW) regions were within the first tertile, demonstrating an unequal distribution of ARI symptoms within those regions. The (NC) and (SS) regions were within the second tertile, showing unequal distribution of ARI symptoms, while (SW) and (SE) regions were within third tertile, indicating a highly unequal distribution of ARI symptoms within those regions. The ***second map*** (green) represents the CI for the year. The (NC), (SE) and (SS) regions were within the first tertile, indicating ARI symptoms are highly concentrated within the low SES groups in these regions. Compared with regions in the first tertile, (NW) region had a medium level of concentration of ARI symptoms among the poor, whereas, (NE) and (SW) regions, showed lower levels of wealth-based inequalities in the distribution of ARI symptoms. The ***third map*** (red) represents tertile scores for prevalence of ARI symptoms across administrative regions. Regions (NW) and (SW) appeared to be within the lowest tertile of prevalence, the (SS) region in the second tertile, and (NE), (NC) and (SE) regions within the highest tertile,─ the region with highest prevalence in ARI symptoms.

## Discussions

Using Gini index and concentration index to explain the sources of the unequal distribution of ARI symptoms prevalence by region and over time especially in the Nigerian context between 2003 to 2013, this study addresses gap in literature regarding region specific determinants that are proximate to children developing ARI symptoms. In the analyses, our study found that prevalence of ARI symptoms has declined between regions and over the years as reported by Nigerian DHS [9–11].

Between regions, variations in the likelihood of ARI symptoms among children existed in the decade under study (2003–2013), although variations appeared to be more evident in 2013. For example, North Central and South Eastern regions displayed non-significant differences in 2003 and 2008, but gained significance in 2013. By the year 2013 Nigeria accounted for high burden of pneumonia deaths [24] also over the decade Nigeria is known to have metamorphorsized into an industrialized country over time due to the oil boom with unequal social and economic development which has also brought about various environmental changes [8, 16]. Further, children residing in North East, South-South and South East regions had a higher likelihood of developing ARI symptoms in the decade. Burgeoning evidence has reported spatial variation in child morbidity, especially the geographic location of the North Eastern regions of Nigeria which is exposed to higher levels of dust exposure, sand storms as it is situated along the Gulf of Guinea trajectory [55]. The higher likelihood in the South-Southern region may reflect children’s exposure to the consequences of environmental degradation due to oil spillage and gas flaring that occurs in the region [16].

**Fig. 1:**
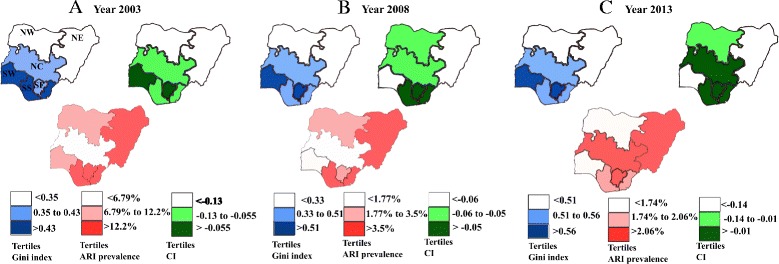
Tertiles of Gini coefficient (map with *blue*), Concentration index (map with *green*) and prevalence of ARI (map with *red*) by region between 2003 and 2013. Regions: NW, North West; NE, North East; NC, North Central; SE, South East; SS, South-South; SW, South West

According to Mosley and Chen [56] on proximate determinants of child survival. In order to better understand the structural determinants influencing proximate factors such as household socio-economic factors, that could explain unequal distribution of ARI symptoms among children; we used (CI) and (GI) to quantify the inequality in the spatial distribution of ARI symptoms while considering the effect of time on its variation. Socioeconomic inequalities in ARI symptoms across household wealth index quintiles were quantified with concentration index [57]. One advantage of using (CI) is that inequalities are assessed across the whole categories of socioeconomic status variable (i.e., wealth index quintiles) rather than only comparing health outcomes in two categories (eg., odds ratios compare one group at a time versus the reference) [58]. As shown in Table 3 and Fig. 1, (CI) was negative and significantly different from zero in most regions, which indicates an excessive concentration of ARI symptoms among children in lowest socioeconomic households. However, the CI for South Western region in 2013 was positive, which indicates children with ARI symptoms were concentrated in socioeconomically better-off families.

While the negative concentration index is consistent with previous literature [35], its positive value in South Western region may indicate that children for educated mothers were more likely to have ARI symptoms [28, 59, 60]. Given that educated mothers are more likely to be from a higher socioeconomic households, educated [59], and mostly be subjected to the menaces of busy road networks due to the heightened development in comparison to other previous years, children are mostly being in care of someone whose lifestyle may influence or exacerbate the likelihood of ARI symptoms. In addition, the educated are more aware of the symptoms and are therefore more likely to report them. Thus, symptom recognition may be differential based on education.

In some regions such as North West and South-South, poorest household wealth index by itself explain persistent wealth-related inequalities in the distribution of ARI symptoms, over the years. According to previous literature carried out in Nigeria, at the advent of socio economic development, the North West and South-South regions in which the morbidity is relatively higher, were not used as the benchmark for free education, agricultural settlements and industrial development unlike the western states/regions [16]. Consequently, parents in these areas are relatively less educated and are less likely to know more about conditions that expose or exacerbate ARI symptoms risk, hence effectively lowering the risk.

Using biomass cooking fuel was the major contributor in North Central [41]. Our finding is in line with a study done in Nigeria which analyzed the national and regional demand and trend between 1971 and 2011 for consumption of biomass energy. Findings reveal firstly a consistent rise in national dependency on biomass fuel over the decades; also a within-regional disparity in the uneven dependency on biomass fuel as the Northern Nigeria has consistently experienced a higher dependency owing to specific drivers such as; availability and cost, poverty level, and cultural preferences [61].

Moreover, we ranked regions based on the results of concentration index tertiles (Fig. 1) and found that between 2003 and 2013, the magnitude of these inequalities remained lowest in North East, and highest in South Eastern region. This indicates in North Eastern region, regardless of the socio economic class a child is from, they are all at risk of developing ARI symptoms. Thus, the high risk of ARI symptoms may be attributed especially to its geographic location along the Gulf of Guinea trajectory as explained earlier that it is most exposed region to higher levels of dust and sand storms [16]. While in the South Eastern region the likelihood of children developing ARI symptoms is related to the socioeconomic inequality over the decade 2003–2013. Burgeoning studies reveal sources of such inequalities arise from context of community infrastructure via policies that affect use of biomass fuel, education, and public health. Thus, living in a community or environment to a large extent partakes equally in the community influences [30]. A study using a standard traditional measurement approach known as the Lorenz curve and Gini co-efficient was used to determine the size of income inequality which reveals a disturbingly growing income inequality in Nigeria since 1991 [62].

Progressively over the years, wealth-related inequalities in ARI symptoms distribution heightened in North Central, North West and South South, whereas ameliorated in South Western region over the decade. Aside the geographic effect, the Northern region has experienced a series of political unrest over the years affecting public health and socio economic development. The presence of the oil industry in the South South region are exposed to oil spillage and these contributes to environmental pollution, and most intricate, wealth gained from the oil boom has led to an increased degradation of the environment and deterioration in the health conditions of many local people [16]. Further, communities in such regions are predominant rural with high levels of social exclusion that face barriers for accessing to adequate health facilities which makes rural area a critical contributor to prevailing wealth related inequalities.

Results could guide interventions aiming at reducing wealth-based inequalities in ARI symptoms by prioritizing its determinants at regional level. For example, risky birth interval and order could be targeted through family planning in regions (eg., North East) where risky birth interval and order explains most of inequalities [37]. In addition, in regions such as the North Central, where cooking methods are also an important contributor, clean cooking options should be promoted. The predictors that contributed 10% or greater to wealth-based inequalities by region in 2013 are presented as follows: In North West*:* poorest household wealth index. In North East: poorest household wealth index, rural residence and risky birth interval. In North Central: poorest household wealth index and biomass cooking methods, In South East: poorest household wealth index, biomass cooking methods and risky birth interval. In South-South: poorest household wealth index, biomass cooking fuel and child’s growth deficit. In South West: poorest household wealth index and risky birth interval.

In Table 4, we have shown the magnitudes and trends of Gini Index to ARI symptoms by year and region. Gini index reflects how much the distribution of ARI likelihood departs from perfectly equal distribution (i.e., zero), thus a region with a value of 0.22 (i.e., North East) deviates less than a region with a value of 0.62 (i.e., South West), this indicate that the likelihood of developing ARI symptoms is more equal in the former than the latter. In the South Western region, wide inequalities were observed across population groups with rural areas which harbor a higher percentage of households with low socio economic status reporting higher numbers of cases than the urban areas. Also, regional inequalities in the development of state exists as development indicators are concentrated in a few Local Government Areas that are urban based [63].

Within the study period, Gini index within regions ranged as follows: North West (0.32–0.34), North East (0.21–0.28), North Central (0.35–0.51), South East (0.38–0.56), South-South (0.33–0.55), South West (0.47–0.62). North Central, South East and South Western regions had a progressive increase in Gini index, while all regions had a perpetual rise between 2008 and 2013, this finding persist when grouping regions according to Gini index tertiles (Fig. 1) [64]. In order to better understand the portion explained by each determinant to unequal distribution of ARI symptoms, we decomposed overall inequalities using relative important methodology [53, 54]. The factors that contributed more than 10% by region in the year 2013 are as follows: In North West: child’s age 6–23 months, child’s gender and type of residence. In North East: child’s age. In North Central: child’s age, cooking method and low maternal education. In South East: child’s age and cooking methods. In South-South: child’s age and risky birth interval. In South West: risky birth interval, child has no health card and child’s age 6–23 months.

This study is not without limitations. First, our measure of ARI symptoms relies on self-reported information. Although DHS quality of data collection is high [9–11, 14, 28, 65, 66], we cannot rule out the occurrence of recall bias. Further, mothers whose children died are more likely to remember more detailed information related to children’s health. In an effort to minimize the potential impact of this bias, we focus on survival children. Second, our findings relied on cross-sectional information, thereby we may not capture the induction time between the onset of overall and wealth-based inequalities and their impact on ARI symptoms.

In the same way, the causal direction between some predictors such as children’s nutritional status, cooking methods, maternal education, household wealth index, or type of residence and ARI symptoms cannot be established. Despite those drawbacks, the present study has various strengths. First, our findings rely on three most recent nationwide representative samples of children below 5 years of age. Second, the relative contribution to wealth-related and overall inequalities are based on regression methods [58]. Third, due to DHS including a similar set of variables across waves, we were able to assess differentials in regional trends as our study is the first to employ the use of the above methodological approach in unraveling socio economic inequalities in ARI symptoms in Nigeria.

## Conclusion

We conclude that despite progress in reduction of ARI over the years, inequality in its distribution remains a public health challenge. Since contributors for overall and wealth related inequalities are not the same, our findings suggest source-specific interventions such as pro-poor interventions eg., maternal education, cleaner cooking fuel, and family planning. Also, because contributors to ARI symptoms differ in each region, our study spearheads information on the supposed specific factors necessary for ARI intervention.

## Appendix

**Table 5:** Descriptive statistics of ARI symptoms prevalence by region, DHS 2003, 2008 and 2013 Nigeria

	North West			North East			North Central			South East			South South			South West		
Variables	2003 *N* = 1517%wt^a^	2008 *N* = 6772%wt^a^	2013 *N* = 8760%wt^a^	2003 *N* = 1234%wt^a^	2008 *N* = 5699%wt^a^	2013 *N* = 5856%wt^a^	2003 *N* = 893%wt^a^	2008 *N* = 4505%wt^a^	2013 *N* = 4286%wt^a^	2003 *N* = 460%wt^a^	2008 *N* = 2160%wt^a^	2013 *N* = 2553%wt^a^	2003 *N* = 482%wt^a^	2008 *N* = 2979%wt^a^	2013 *N* = 3498%wt^a^	2003 *N* = 573%wt^a^	2008 *N* = 3084%wt^a^	2013 *N* = 3643%wt^a^
Child’s gender (female)																		
Female	8.52	2.20	0.76	17.10	7.33	5.48	5.72	1.03	2.37	7.65	1.80	1.92	11.4	3.00	1.83	6.55	0.90	0.70
Male	9.19	1.62	0.96	15.42	7.76	4.76	7.79	1.51	1.76	4.70	1.75	2.19	13.14	3.97	1.65	6.99	1.14	0.90
Child’s age (months)																		
0–5	14.68	1.80	0.46	18.99	4.78	4.93	6.88	1.43	1.23	4.36	1.51	1.84	13.53	2.48	0.82	2.47	0.69	0.00
6–11	12.59	2.06	0.95	28.62	10.41	7.00	9.85	1.83	3.28	11.77	2.44	4.62	21.69	3.40	2.36	6.35	0.98	1.92
12–23	11.80	2.65	1.28	13.96	10.10	7.68	8.84	1.94	4.21	12.11	2.94	3.44	13.13	4.96	2.98	9.05	1.39	0.94
24–35	9.87	2.17	1.11	16.94	7.37	5.25	6.44	1.05	1.88	4.15	1.38	1.72	9.07	4.96	2.07	5.06	1.72	1.21
36–59	3.49	1.37	0.61	11.99	6.46	3.26	4.59	1.15	0.96	3.91	1.22	0.65	9.95	2.32	0.90	8.31	0.63	0.42
Birth order and interval																		
1st birth	10.74	1.92	0.71	18.79	6.59	3.39	7.06	1.16	2.09	4.02	2.17	2.76	17.32	3.75	2.39	4.44	1.34	0.88
2 to 4 and short	9.83	1.10	0.65	19.13	7.96	4.68	9.81	1.23	1.36	1.32	1.10	1.10	14.01	1.44	0.15	7.55	0.36	3.05
2 to 4 and long	9.82	2.15	1.02	14.89	6.05	4.44	4.36	1.27	2.29	9.78	2.00	1.70	12.04	3.48	2.02	6.44	1.28	0.54
5 to + and short	2.38	2.77	0.89	17.89	8.56	6.37	1.94	2.71	2.17	7.51	0.88	3.02	23.97	5.59	0.39	9.88	0.66	0.00
5 to + and long	8.25	1.67	0.82	14.51	9.04	6.56	9.47	1.49	1.91	8.76	1.91	2.29	5.53	3.77	1.67	10.53	0.20	0.91
Height/Age (chronic)																		
Deficit	7.65	1.98	0.85	12.50	8.70	5.95	6.11	1.48	1.81	5.62	1.29	0.63	22.63	5.57	3.93	6.36	1.57	1.20
Normal	10.33	1.98	0.80	19.75	6.66	5.05	8.71	1.55	2.18	6.94	1.48	2.53	10.92	3.58	1.45	6.68	1.01	0.77
Above normal	16.09	1.43	3.06	28.44	10.76	1.10	9.47	0.40	2.22	3.40	4.54	0.41	11.71	3.58	0.41	0.00	0.00	0.00
Received BCG																		
Yes	8.14	2.56	0.72	18.16	6.10	5.62	8.35	1.60	2.07	6.40	1.96	2.08	11.33	3.17	1.80	6.12	1.02	0.87
No	9.15	1.71	0.90	15.77	8.04	4.85	4.14	1.13	2.00	6.42	1.02	1.99	15.34	4.56	1.48	11.23	1.09	0.51
Don’t know	8.89	0.00	0.00	0.00	17.33	8.40	26.06	0.00	7.72	5.06	0.00	0.00	0.00	0.00	0.00	0.00	0.00	0.00
Child has health Card																		
No	8.55	1.88	0.86	15.84	8.31	4.65	5.37	1.73	2.17	4.99	1.25	1.89	17.30	5.15	1.93	9.50	1.43	0.92
Yes (seen/no seen)	10.60	2.04	0.81	17.24	5.62	5.89	7.88	1.09	1.83	6.53	1.94	2.11	9.16	3.03	1.78	5.25	0.96	0.78
No longer has card	10.68	2.30	1.77	13.98	7.00	6.92	7.71	1.41	3.81	3.59	0.00	0.00	8.15	0.40	0.48	19.18	0.85	0.00
Household Characteristics																		
Cooking methods																		
Kerosene	12.95	1.93	0.16	13.71	15.91	4.39	3.24	0.39	0.65	4.44	1.29	0.67	14.51	2.78	1.57	4.83	1.05	0.59
Biomass	8.46	1.93	0.92	16.45	7.32	5.20	7.35	1.56	2.34	9.41	2.03	2.75	12.60	4.09	1.71	10.61	1.05	1.44
Others	2.85	0.00	0.00	4.46	9.96	1.88	3.16	0.00	0.79	4.41	0.00	0.00	0.00	0.00	2.75	11.97	0.00	0.00
Maternal education																		
No education	8.99	2.09	0.95	15.18	7.73	5.03	7.37	1.32	0.86	16.26	3.29	5.33	14.20	0.96	0.86	11.59	2.16	0.11
Primary	9.49	1.26	0.58	16.06	8.07	6.03	7.52	1.53	3.01	6.43	2.07	1.04	17.72	4.44	1.38	8.19	0.60	1.46
Secondary	7.98	1.51	0.65	22.93	4..39	4.98	5.15	1.31	3.49	6.03	1.45	2.24	7.79	3.57	1.70	5.41	1.11	0.86
Higher	4.49	0.00	0.00	21.09	12.66	2.92	0.00	1.46	0.34	1.65	1.49	2.27	8.75	1.04	3.40	2.34	0.36	0.00
Household wealth index																		
Poorest	8.46	2.14	1.05	13.44	7.26	4.70	7.31	1.02	0.97	16.11	2.00	0.46	13.61	8.54	0.00	13.79	0.69	0.58
Poorer	10.45	2.40	1.18	19.78	9.00	6.42	3.89	1.97	4.28	8.74	3.29	3.78	14.25	4.59	2.68	11.77	1.19	0.66
Middle	9.44	1.21	0.52	17.72	6.25	5.46	8.50	1.54	2.11	7.69	2.54	3.27	18.44	3.12	1.59	10.75	1.68	0.74
Richer	6.43	1.07	0.25	16.57	7.93	4.03	8.44	1.33	0.79	3.81	0.86	2.14	10.78	3.36	1.54	6.04	1.09	0.92
Richest	8.78	1.71	0.00	11.43	5.85	2.98	3.30	0.68	1.06	4.67	1.47	0.58	7.34	1.91	1.77	4.80	0.79	0.79
Type of residence																		
Urban	8.69	1.32	0.36	14.69	7.56	4.16	4.42	1.03	1.23	4.09	1.67	2.19	6.77	2.76	2.02	4.33	0.95	0.70
Rural	8.93	2.04	1.01	16.75	7.54	5.43	7.40	1.51	2.31	8.59	1.86	1.76	14.13	3.78	1.59	12.28	1.11	1.07
Outcome measure																		
Symptoms of ARI	8.9	1.9	0.9	16.2	7.5	5.1	6.8	1.4	2.06	6.4	1.8	2.1	12.2	3.5	1.7	6.8	1.0	0.8

^a^Weighted percentages and means are reported. Percentages may not add up to 100 due to rounding
